# Organization of prefrontal network activity by respiration-related oscillations

**DOI:** 10.1038/srep45508

**Published:** 2017-03-28

**Authors:** Jonatan Biskamp, Marlene Bartos, Jonas-Frederic Sauer

**Affiliations:** 1 Physiologisches Institut I, Systemic and Cellular Neurophysiology, Albert-Ludwigs-Universität Freiburg, Hermann-Herder-Straße 7, 79104 Freiburg, Germany

## Abstract

The medial prefrontal cortex (mPFC) integrates information from cortical and sub-cortical areas and contributes to the planning and initiation of behaviour. A potential mechanism for signal integration in the mPFC lies in the synchronization of neuronal discharges by theta (6–12 Hz) activity patterns. Here we show, using *in vivo* local field potential (LFP) and single-unit recordings from awake mice, that prominent oscillations in the sub-theta frequency band (1–5 Hz) emerge during awake immobility in the mPFC. These oscillation patterns are distinct from but phase-locked to hippocampal theta activity and occur synchronized with nasal respiration (hence termed prefrontal respiration rhythm [PRR]). PRR activity modulates the amplitude of prefrontal gamma rhythms with greater efficacy than theta oscillations. Furthermore, single-unit discharges of putative pyramidal cells and GABAergic interneurons are entrained by prefrontal PRR and nasal respiration. Our data thus suggest that PRR activity contributes to information processing in the prefrontal neuronal network.

Theta oscillations provide a temporal reference for multimodal signal integration in neurons and are therefore thought to support information processing in cortical networks^1^,^2^. These activity patterns are generated in hippocampal-septal loops^3^,^4^ and are transmitted to the mPFC through the ventral portion of the hippocampus^5^,^6^. In the PFC, theta oscillations have been linked to cognitive processes including working memory^2^,^7^ and memory recollection^8^,^9^. It has been suggested that these functions are realized by the transient synchronization of principal cell assemblies by theta-modulated gamma rhythms^10^,^11^.

However, this ‘theta theory’ contains some inconsistencies. The power of hippocampal theta activity, which drives theta oscillations in the mPFC, depends on locomotion and is attenuated when animals remain immobile^12^,^13^,^14^,^15^ while prefrontal functions remain intact in the absence of motion. Furthermore, the amplitude of prefrontal theta oscillations has been reported to be small compared to the hippocampus, showing only transient ‘bursts’ of high power^16^,^17^. These considerations led us to hypothesize that the ‘theta hypothesis’ as the underlying mechanism of information processing in prefrontal circuits should be revisited.

Recently, a novel slow respiration-locked rhythm (RR) has been identified in the hippocampus^18^,^19^,^20^ and barrel cortex of mice^21^. Interestingly, hippocampal RR prominently emerges during awake immobility and entrains discharges of subpopulations of neurons, suggesting that this activity pattern may contribute to information processing in cortical neuronal circuits, especially when theta activities are weak^20^. On the basis of these observations, we hypothesized that RRs might functionally complement theta patterns, particularly during immobility, and play a role in the organization of prefrontal network activity. We addressed this question using *in vivo* LFP and single-unit recordings from the mPFC of awake mice.

## Results

### Respiration-coupled LFP oscillations emerge in the mPFC of awake mice

**Figure 1:**
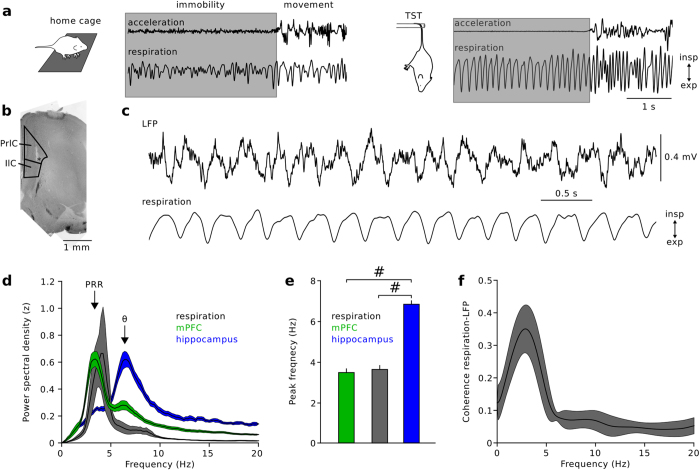
Respiration-related LFP oscillations in the medial prefrontal cortex of awake mice. (**a**) Recording respiration with a nasal thermocouple during free home cage behaviour (left) and tail suspension test (TST, right) revealed stable respiration frequency during immobility in the TST. The traces on top display movement extracted from the reading of an accelerometer mounted on the recording amplifier. Grey areas correspond to immobile epochs. (**b**) LFP recordings were targeted to the prelimbic (PrlC) and infralimbic (IlC) portions of the mPFC. Asterisk denotes the location of the recording electrode. (**c**) Example LFP (1–200 Hz-filtered, top) and respiration recording (1–10 Hz-filtered, bottom) during TST immobility. Inspiration faces upwards. (**d**) Power spectral density analysis revealed prefrontal respiration rhythms (PRRs, green) that spectrally overlap with respiration (grey) but not with theta activity in the mPFC (small peak at ~7 Hz) or hippocampus (blue). (**e**) Summary plot of peak frequency of prefrontal LFP (green, n = 25 mice), respiration (grey, n = 4) and hippocampal LFP (blue, n = 13). Mann-Whitney U-tests with Bonferroni correction. (**f**) Prefrontal LFPs are highly coherent with nasal respiration (n = 4 mice). Data are mean ± sem. ^#^p < 0.001.

To explore the potential occurrence of RR activity in the mPFC, we recorded LFPs from layer 5 of the prelimbic as well as infralimbic cortices of mice while simultaneously monitoring nasal respiration with a thermocouple implanted into the nasal cavity (Fig. 1a,b). The spectral characteristics of the LFP were indistinguishable between prelimbic and infralimbic recording sites, so the data were pooled (Supplementary Fig. S1). Immobile periods were visually identified based on video tracking of the animals’ paths (n = 16 mice) or based on data acquired with an accelerometer mounted on the recording amplifier (n = 9 mice; Fig. 1a). In agreement with previous reports, breathing frequency was highly variable during free movement in the home cage^20^ (Fig. 1a). Subjecting mice to a tail suspension test^22^ (TST), in which the animals alternate between epochs of escape behaviour and purely passive coping, resulted in highly regular breathing patterns, particularly during motionless epochs (Fig. 1a). Repeated exposure to TST did not alter spectral properties or behavioural performance (Supplementary Fig. S2). Based on these observations, we focussed our analysis on immobile epochs during the TST. However, qualitatively similar findings were obtained during home cage behaviour (summarised in Supplementary Fig. S3).

During TST immobility, prefrontal LFPs displayed a prominent power peak at 3.50 ± 0.19 Hz (n = 25), which overlapped with the power peak of respiration (peak frequency 3.65 ± 0.19 Hz, n = 4, p = 0.328 for the comparison of LFP and respiration peak frequencies, Mann-Whitney U-test; Fig. 1c,d; Supplementary Fig. S4) but was spectrally distinct from hippocampal theta (peak frequency 6.68 ± 0.17 Hz, n = 13, p = 1 * 10^−6^ and 2 * 10^−7^ for the comparisons to prefrontal and respiration peak power frequency, respectively; Mann-Whitney U-tests; Fig. 1d,e). The prefrontal LFP was highly coherent with nasal respiration (peak coherence 0.37 ± 0.067, frequency of highest coherence: 2.80 ± 0.40 Hz, n = 4; Fig. 1f). Thus, respiration-locked oscillations emerge in the mPFC during immobile epochs. We termed this activity pattern *prefrontal respiration rhythm* (PRR).

**Figure 2:**
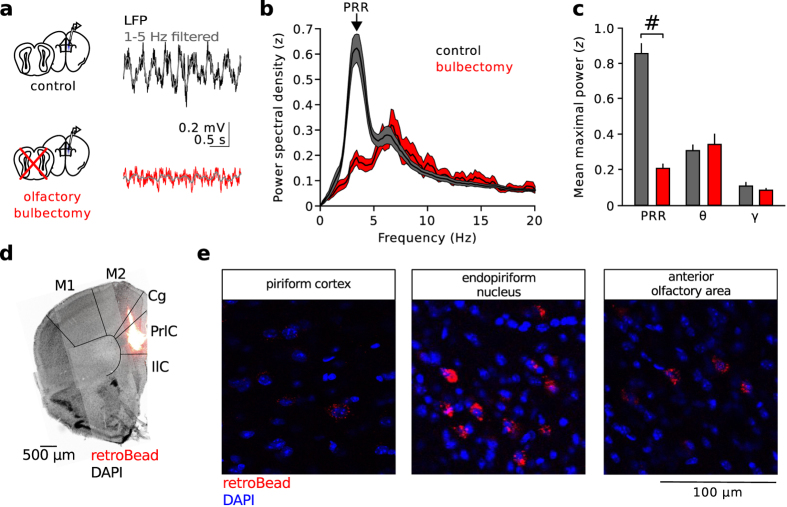
PRR activity requires intact olfactory bulbs. (**a**) Example LFP traces recorded after bilateral bulbectomy (red, bottom) and under control conditions (grey, top). Grey superimposed traces are 1–5 Hz filtered to emphasize PRR activity. (**b**) Power spectral density of prefrontal LFPs after bulbectomy reveals a reduction in PRR power (1–5 Hz). (**c**) Summary graph of PRR (1–5 Hz), theta (6–12 Hz), and gamma power (30–100 Hz) after bulbectomy (red) and under control conditions (grey). Bulbectomy strongly reduces PRR (unpaired *t-*test) but leaves theta and gamma power unaffected (Mann-Whitney U-tests). (**d**) Frontal slice of the mPFC shows the area of red retroBead injection for the retrograde tracing of mPFC inputs. Cg: cingulate cortex, IlC: infralimbic cortex, PrlC: prelimbic cortex, M1, 2: primary and secondary motor cortex. (**e**) Retrogradely traced cells (red) were detected in olfactory cortices. Blue: DAPI signal. Representative for 3 mice. ^#^p < 0.001. Data represent mean ± sem.

Hippocampal RR activity is driven by respiration-entrained oscillatory input from the olfactory bulb (OB). To test whether the OB is required for the emergence of PRR, we surgically removed both OBs and examined prefrontal LFPs (Fig. 2). Bilateral bulbectomy resulted in a strong reduction of PRR power (measured in the 1–5 Hz frequency band, p = 1.4 * 10^−6^, n = 7 bulbectomized and 25 control mice, unpaired *t*-test; Fig. 2a–c) while the power of theta or gamma oscillations (p = 0.494 and p = 0.964, respectively; Mann-Whitney U-tests; Fig. 2c) as well as the proportion of time spent freezing during the TST remained unchanged (Supplementary Fig. S5). To identify the potential anatomical basis of projections from the olfactory system to the mPFC, we injected the retrograde tracer redRetroBead into the mPFC (Fig. 2d). Histological processing of the PFC revealed high spatial specificity of the injection site in the prelimbic and cingulate area of the mPFC (Fig. 2d). Retrogradely labelled cell bodies were found in relay areas of the olfactory system, which have been shown to receive inputs from the OB^23^,^24^. Labelled cells were detected in the piriform cortex (5.5 ± 2.8% of DAPI-positive cells showed detectable levels of retroBead, n = 3 mice), dorsal endopiriform nucleus (11.4 ± 4.5% retrogradely traced cell bodies, n = 3 mice) and anterior olfactory area (14 and 2% retroBead-positive cells, 2 mice; Fig. 2e). In addition, labelled somata were found in other input regions of the mPFC, including the ventral hippocampus and ventro-orbital cortex (Supplementary Fig. S6). Taken together, our data suggest that RR activity is transmitted through the OB. Furthermore, we confirmed the presence of anatomical routes from olfactory relay areas onto neurons in the mPFC, which might participate in the propagation of respiration-related LFP oscillations to the mPFC.

### PRR activity exhibits phase-phase coupling with hippocampal theta

**Figure 3:**
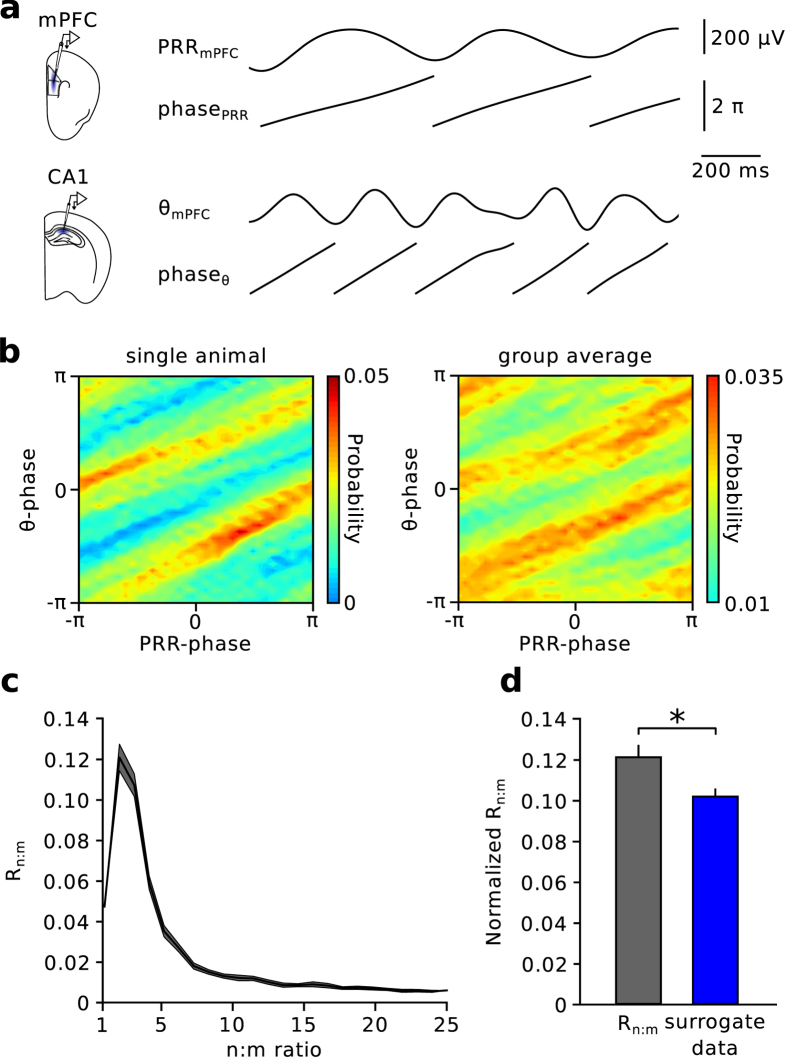
Phase-phase coupling of PRR with hippocampal theta. (**a**) Simultaneous recording of prefrontal and hippocampal LFPs. Traces show frequency-filtered signals (PRR: 1–5 Hz, theta: 6–12 Hz) and the respective instantaneous phases obtained by Hilbert transformation. (**b**) Joint phase histograms of hippocampal theta phase as a function of PRR phase revealed diagonal stripes of increased probability. Left: data from one mouse. Right: Average of 12 mice. (**c**) R_n:m_ phase-phase coupling analysis revealed a maximum at *m* = 2, suggesting that two cycles of hippocampal theta occur within one cycle of PRR. (**d**) Statistical comparison to surrogate data (time-shifted by a random interval between 5 and 10 s) indicated significant phase-phase coupling (paired *t-*test). *p < 0.05. Data represent mean ± sem.

The PFC receives theta-modulated hippocampal input, which is thought to support prefrontal functions including the execution of working memory^2^. Having established the emergence of PRR activity in the mPFC, we investigated whether PRR activity occurs phase-locked to theta oscillations (Fig. 3). Histograms of PRR phase as a function of hippocampal theta phase revealed diagonal stripes of enhanced probability, suggesting phase-phase coupling between both oscillators (Fig. 3b).

To statistically assess phase-phase coupling between PRR and theta, we employed surrogate methods that allow to distinguish actual from spurious coupling results^25^. We measured the phase consistency between theta and PRR while systematically accelerating PRR phase by a factor *m* (see Materials and Methods). The resulting metric, *R*_*n:m*_, varies for each *m* between 0 (no phase locking) and 1 (perfect locking) and can be statistically compared to appropriate surrogate vectors^25^ (see Materials and Methods). R_n:m_ analysis revealed maximal phase locking between PRR and hippocampal theta at *m* = 2 (Fig. 3c). Statistical comparison to surrogate data indicated mild but significant coupling (p = 0.023, n = 12 mice, paired *t*-test; Fig. 3d), suggesting a 2:1 phase-phase relationship between theta and PRR. Thus, PRR and hippocampal theta oscillations occur phase-coupled to each other.

### PRR activity modulates fast gamma oscillations

**Figure 4:**
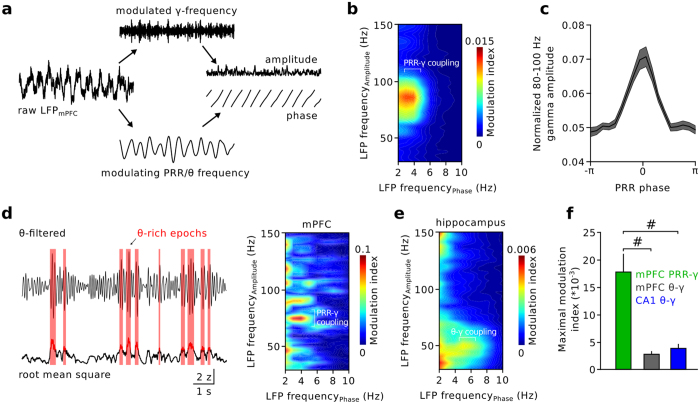
PRR activity modulates the amplitude of prefrontal gamma oscillations. (**a**) Schematic illustration of the analysis. The phase of the slow modulating PRR or theta oscillation and the amplitude of the fast modulated gamma frequency components were obtained by Hilbert transformation. (**b**) Modulation of the amplitudes of gamma frequency activity patterns (30–100 Hz) by the phase of ongoing PRR and theta frequency oscillations during TST immobility. The colour code indicates modulation depth, quantified as a modulation index (0: no modulation, 1: maximal modulation, see Methods). Average of 25 mice. (**c**) Gamma oscillation amplitude is enriched at the peak of RR phase. Zero denotes the peak, −π and π the troughs of the PRR cycle. (**d**) Restricting the analysis to time epochs of strong theta activity (red areas on the left, see Methods) still revealed prominent PRR-gamma coupling. n = 12 mice. (**e**) Gamma oscillations were modulated by theta activity in the hippocampus (CA1). n = 13 mice. (**f**) Summary of gamma amplitude modulation by PRR and prefrontal as well as hippocampal theta. Wilcoxon signed-rank and Mann-Whitney U-tests with Bonferroni correction. ^#^p < 0.001. Data represent mean ± sem.

Entrainment of fast gamma oscillations by slower neuronal network rhythms (e.g. theta) is thought to support the formation and synchronization of co-active cell assemblies^11^,^26^. We therefore asked whether PRR activity may entrain local gamma oscillations in a similar fashion as previously demonstrated for theta oscillations^27^,^28^ (Fig. 4). We determined the phase-amplitude modulation of gamma oscillations by ongoing PRR^29^ (Fig. 4a). This analysis revealed a prominent modulation of high-gamma amplitude (80–100 Hz) by PRR phase (Fig. 4b). High-gamma oscillations occurred phase-locked to the peak of PRR activity (n = 25 mice; Fig. 4c). PRR-gamma coupling in the mPFC was significantly stronger than theta-gamma coupling (modulation index_PRR_ 0.018 ± 0.003 versus modulation index_theta_ 0.003 ± 0.001, p = 0.00001, n = 25 mice, Wilcoxon signed-rank test; Fig. 4b,f), even when we restricted our analysis to epochs of pronounced theta activity (defined as the root mean square of the theta-filtered signal exceeding the mean plus two standard deviations, ratio of PRR-gamma coupling and theta-gamma coupling: 4.51 ± 1.12, analysis performed for n = 12 mice; Fig. 4d). Furthermore, PRR-gamma coupling in the mPFC was significantly stronger than theta-gamma coupling in the hippocampus (modulation index_theta_ 0.004 ± 0.001, n = 13, p = 0.0003, Mann-Whitney U-test; Fig. 4e,f; note that hippocampal theta-gamma coupling did not require the animal to be moving, Supplementary Fig. S7). Thus, prefrontal PRR patterns efficiently entrain fast gamma oscillation amplitude.

### PRR activity entrains prefrontal single-unit firing

**Figure 5:**
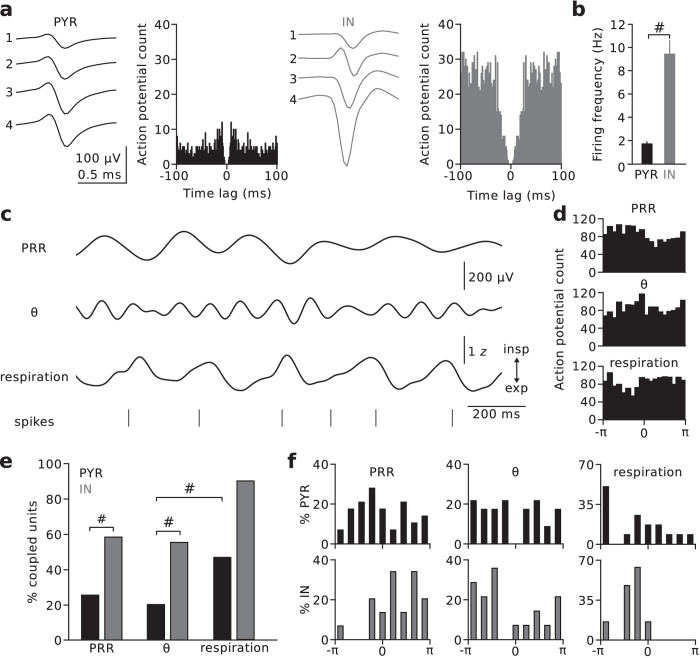
PRR activity entrains prefrontal single-unit activity. (**a**) Single-units were recorded with stereotrodes or tetrodes lowered to the mPFC on a microdrive (n = 224 units). Examples show average waveforms of the four channels of a tetrode recording and the respective autocorrelation function of a putative pyramidal cell (PYR, left) and a putative GABAergic interneuron (IN, right). (**b**) INs discharged at significantly higher frequency than PYRs. Mann-Whitney U-test. (**c**) Example of PRR-filtered LFP (1-5 Hz), theta-filtered LFP (6-12 Hz), simultaneously recorded nasal respiration, and spiking activity of a PYR unit (bottom). (**d**) Spike histograms of the unit shown in (**c**) as a function of PRR, theta, and respiration phase indicated significant coupling of the discharge activity to the various rhythms (Rayleigh’s test, p < 0.05). (**e**) Summary graph of the percentage of significantly coupled PYR (black) and IN units (grey). Chisquare and Fisher’s exact tests with Bonferroni correction. (**f**) Summary histograms of the preferred phase of all significantly phase-coupled PYR (black, top) and IN units (grey, bottom) to the three types of rhythms. Zero denotes the peak, −π and π the troughs of the oscillation cycles. ^#^p < 0.001. Data represent mean ± sem.

To investigate whether prefrontal neuronal activity is modulated by PRR activity, we recorded prefrontal single-units (n = 224 single units from 7 mice; Fig. 5a). Putative pyramidal neurons (n = 187) and GABAergic interneurons (INs; n = 37) were identified based on the waveform shape of single-units^30^ (INs had shorter half-durations and faster trough-to-peak times than pyramidal units; spike half-duration: IN 0.25 ± 0.02 ms versus pyramidal units 0.47 ± 0.01 ms, p = 4 * 10^−12^; spike trough-to-peak time: IN 0.20 ± 0.01 ms versus pyramidal units 0.31 ± 0.01, p = 5 * 10^−6^, Mann-Whitney U-tests; Supplementary Fig. S8). Consistent with previous reports, INs discharged action potentials at significantly higher frequency than pyramidal units^17^,^31^ (IN: 9.4 ± 1.2 Hz, pyramidal: 1.7 ± 0.2 Hz, p = 2 * 10^−12^, Mann-Whitney U-test; Fig. 5b).

During immobility in the TST, a substantial proportion of pyramidal units (~26%, 41 out of 161 with sufficient spiking activity during immobility, see Materials and Methods) and INs (21 out of 36, ~58%) discharged action potentials significantly phase-locked to PRR (p < 0.05, Rayleigh’s test for circular uniformity; Fig. 5c–e). A similar proportion of pyramidal (33 out of 161, ~21%) and IN units (20 out of 36) fired phase-locked to theta activity (Fig. 5c–e). A significantly higher proportion of INs than pyramidal units was entrained by PRR (p = 0.0001; Fig. 5c) and theta activity (p = 0.00002, Chisquare tests with Bonferroni correction, p_critical_ = 0.0056; Fig. 5c–e). In case of pyramidal units, ~61% of PRR-coupled and ~52% of theta-coupled neurons were also entrained by theta and PRR, respectively. In case of INs, the percentage of units coupled to both oscillation types was 38% and 35%. PRR- and theta-coupled units thus comprised partially overlapping populations.

The preferred phases of significantly modulated units followed a broad distribution with the highest number of pyramidal units modulated by the late ascending phase of the PRR cycle (circular mean −0.89 with −π and π denoting the troughs and zero indicating the peak) and the ascending and descending phases of theta (Fig. 5f). Most INs, in contrast, exhibited phase-locked discharges during the descending phase of PRR (circular mean: 1.34) and the ascending phase of theta cycles (Fig. 5f).

Finally, we investigated whether mPFC units discharges were directly entrained by nasal respiration (41 pyramidal and 10 INs from 2 mice were recorded with simultaneous acquisition of respiration; 36 of the pyramidal units displayed sufficient spiking activity during immobility, see Materials and Methods; Fig. 5c–e). ~47% of pyramidal units (17 out of 36) and 90% of INs (9 out of 10) discharged phase-locked to respiration (Fig. 5e). The highest number of pyramidal units were entrained around the peak of expiration while most INs fired action potentials time-locked to the rising phase of inspiration, which roughly reflected the PRR-coupling phase difference of ~2 between pyramidal and IN units (Fig. 5f). There was substantial overlap in coupling to PRR and respiration: Out of 17 respiration-coupled pyramidal units, 8 were also entrained by PRR activity. This overlap was even more pronounced for the IN population. 8 out of 9 respiration-entrained INs fired action potentials coupled to PRR activity. Jointly our data indicate that respiration and PRR substantially contribute to the temporal organization of unit activity in the mPFC.

## Discussion

We have identified a respiration-entrained oscillation pattern distinct from theta in the mPFC of awake mice (Fig. 1). PRR activities entrain local gamma oscillations and single unit discharges (Figs 4 and 5), suggesting that PRR contributes to the processing of multimodal information streams that reach the mPFC from cortical and subcortical brain areas. Although RRs have been shown to emerge in the hippocampus^20^ and barrel cortex^21^ of awake, the piriform cortex of ketamine-anesthetized mice^32^, and the piriform cortex of humans^33^, we provide first evidence that the mPFC, a key associational brain region, is paced by the respiration cycle. We propose that this prefrontal rhythm may support the synthesis of multimodal information content as a basis of decision making and execution of behaviour, particularly during motionlessness. Furthermore, phase-synchrony of PRR with hippocampal theta activity may be a mechanism for linking the decision making circuitry with the spatial-contextual system.

For the meaningful interpretation of LFP recordings it is essential to tell apart local from volume-conducted signals^6^. Gamma oscillations, which are considered to be spatially constrained, as well as local prefrontal unit discharges were phase-modulated by PRR. These findings suggest that PRR activity reflects a local phenomenon rather than volume conduction from elsewhere in the brain. Another important aspect is to distinguish PRR activities from type II theta (also referred to as atropine-sensitive theta). Although not tested directly in this study, we propose that the observed PRR is distinct from type II theta because PRRs were paced by respiration. Furthermore, RR activity in the hippocampus has been shown to be insensitive to atropine.

How might the coupling between respiration and electrical oscillations in the prefrontal LFP be achieved? Olfactory sensory neurons possess mechanosensitive properties^34^. Airflow through the nose might therefore excite sensory neurons which synapse on mitral and tufted cells, the main output neurons of the OB. Nasal airflow is indeed required for RR activities to emerge in the barrel cortex^21^ and hippocampus^18^ of mice as well as in the piriform cortex of humans^33^. In anaesthetized mice and rats, discharge activity of ~50–65% of mitral and tufted cells has been shown to be significantly entrained by the respiration cycle^35^,^36^. Rhythmic activity of OB neurons is transmitted to downstream targets. Activity in the piriform cortex, which receives direct input from the OB^24^, has been demonstrated to be linked to the respiration rhythm in ketamine-anesthetized mice^32^ and human participants^33^. Moreover, the membrane potential of piriform pyramidal units rhythmically fluctuates aligned with the breathing rhythm^32^. Our retrograde tracing analysis indicates that neurons in the olfactory cortices, including the piriform cortex, contact prefrontal pyramidal neurons (see also ref. 37). It is therefore plausible that nasal air movement is converted into discharge activity in the OB and then transmitted to the mPFC via olfactory cortical regions.

What might be the functional role of PRR activity in the mPFC? The mPFC receives and associates information from various cortical and subcortical areas and is involved in the planning and execution of behaviour. Experimental and theoretical studies indicated that theta-modulated gamma rhythms are important for the synchronization of principal cell assemblies and might thereby underlie the encoding and representation of information as the basis of cognitive processes including working memory^2^,^7^ and memory recollection^8^,^9^. We propose that entrainment of prefrontal gamma activity and neuronal discharges by PRR may fulfil similar functions, particularly when theta activities are weak. Future efforts should examine the functional role of PRR activity on the cognitive level.

Recent work has identified slow oscillations in a similar frequency range as PRR. So called 4 Hz activity patterns are capable of entraining gamma oscillations and neuronal discharges in the PFC. They have been observed during the working memory component of a spatial working memory task in rats^38^. The 4 Hz rhythm is most likely independent of respiration because breathing frequency is highly variable during epochs of movement^20^ (when 4 Hz appears). Similarly, 4 Hz oscillations have been described to pace prefrontal neuronal assemblies during fear-induced freezing^39^,^40^. Although not tested directly in that study, it is possible that freezing-associated 4 Hz patterns are linked with respiration. Based on those findings, we propose that PRR is a member of a family of sub-theta frequency activity patterns which emerge during different behavioural states including working memory^38^ (4 Hz rhythm), anxiety^39^,^40^ (4 Hz rhythm), and wake immobility^20^ (this study). These activity patterns might serve the purpose of synchronizing prefrontal neuron discharges and link them with oscillation activity in distant brain regions (e.g. hippocampal theta). Further studies are required to examine the neuronal networks underlying the generation of the various low frequency oscillation patterns in prefrontal circuits. Addressing these questions will improve our understanding of the mechanisms supporting information processing in the mPFC.

## Methods

### Animals

C57/Bl6 mice aged 6–18 postnatal weeks (http://jaxmice.jax.org/strain/013636.html) of both sexes were used in this study. Experimental protocols were approved by the ‘Tierversuchskommission’ of the Regierungspräsidium Freiburg (Approval number G16-102) in accordance with national legislation. Animals were housed in groups under a 12 h dark-light cycle. After implantation of electrodes (see below), the mice were housed individually for 2–21 days.

### Electrode implantations

LFP recordings were performed in the mPFC and the hippocampus of freely moving mice. The electrodes were chronically implanted in the mPFC (n = 18 mice), in the dorsal CA1 of the hippocampus (n = 13 mice, 12 of which also had an mPFC electrode). Seven mice were implanted with movable arrays of 3–6 stereotrodes or tetrodes mounted on a microdrive following published designs^41^ to obtain single-unit recordings in the mPFC (see below). Two mice with chronic mPFC electrodes and two mice with microdrives had additional thermocouples implanted into the nasal cavity to monitor respiration. After the recording, electrode locations were histologically verified (see below).

The animals were anaesthetized with isoflurane (induction 3%, maintenance 1–2% in O_2_ at an airflow of 1–2 l/min) and fixed in a stereotaxic frame (Kopf Instruments). Buprenorphine was injected subcutaneously during the surgery (0.05–0.1 mg/kg body weight). Body temperature was kept stable with a heating pad (38 °C, Witte & Sutor GmbH). Craniotomies (1–2 mm diameter) were performed to implant stainless-steel wire electrodes of 125 μm diameter according to stereotaxic coordinates (right mPFC: 1.9 mm anterior to bregma, 0.7 mm lateral to the midline, and 2 mm below the brain surface at a 10° angle, or 1.9 mm anterior to bregma, 0.45 mm lateral to the midline, and 2.5 mm ventral to bregma; right dorsal CA1: 2.0 mm posterior, 1.5 mm lateral, and 1.55–1.60 mm ventral of bregma). For bulbectomy, a craniotomy was performed above the OB (4.5 mm anterior, 0.6 mm lateral, and 1.6–2 mm ventral of bregma). OBs were removed by aspiration using a blunt 23-gauge syringe needle connected to a vacuum pump (Aqua Medic). Recordings from bulbectomized mice were performed after a recovery period of 2 days to avoid confounds by long-term changes induced by anosmia^42^,^43^. Microdrives holding stereotrodes or tetrodes fabricated from Ni-chrome wire (12.5 μm diameter, California Fine Wire Company) were implanted over the mPFC (1.9 mm anterior, 0.45 mm lateral, and 1.5–1.9 mm ventral of bregma). The recording electrodes were gold-plated to a final impedance of 150–350 kΩ and lowered to the mPFC at a speed of ~100–150 μm/day over the course of 4–7 days. Reference and ground wires were connected to stainless steel screws (1 mm diameter) in the bone over the cerebellum. A thermocouple (80 μm diameter, Omega Engineering part 5TC-TT-KI-40-1M) was implanted into the right nasal cavity (11 mm anterior, 0.5 mm lateral of bregma). Dental cement was used to fix the electrodes and thermocouple in place.

### Tracer injection

Injection of red RetroBeads (1:1 in sterile-filtered PBS, 0.5 μl, unilateral injection; Lumafluor) into the right mPFC (anterior: 1.95 mm, lateral: 0.45 mm, ventral: 2.5 mm from bregma, n = 3 mice) was performed with a Hamilton syringe at an infusion rate of ~0.1 μl/min. Three days after injection, the animals were intracardially perfused and the brains sectioned (see below). Image analysis was performed with a confocal microscope (Zeiss LSM710).

### Behavioural testing and analysis

All behavioural experiments were carried out in a sound-attenuating chamber. For home cage recording, the cage was placed inside the sound-attenuating chamber and monitored with an overhead camera (TP-link) for 6–10 min/day. Up to 5 recording sessions (one recording session per day) were preformed. For the TST, the mice were fixed to a horizontal bar at ~25 cm height. Movement was digitally recorded from the side for 6–9 min once per day on up to 5 subsequent days. Periods of immobility during home cage and TST recording were visually defined as the complete absence of motion (except for breathing) and were identified offline from video files or from accelerometer recordings (see Fig. 1).

### *In vivo* electrophysiology

LFPs were recorded with a wireless amplifier system (W4, Multichannel Systems) 2–21 days after surgery at a sampling rate of 2 kHz or with a wired recording system (RHD2000, Intan Technologies, sampling rate 10 kHz). Single-unit activity was recorded at 20–30 kHz using a 32-channel amplifier system (RHD2000, Intan Technologies). After the last recording session, the animals were deeply anaesthetized with an intraperitoneal injection of urethane (2 g/kg). To identify recording locations, electrolytic lesions were made by briefly (~1 s) applying 10–20 V to each electrode. The animals were intracardially perfused with phosphate-buffered saline (~1 min) followed by 4% paraformaldehyde (~10 min). The brains were sectioned (slice thickness 100–200 μm) and inspected with a light microscope. A subset of brains were stained with cresyl violet or 4′,6′-diamidino-2-phenylindole. Only recording areas located in the prelimbic or infralimbic cortex of the mPFC and in the stratum pyramidale or oriens of CA1 were accepted for analysis.

### Data analysis

LFP data were analysed with build-in and custom-made routines running in the Python 2.7 Spyder IDE. The raw LFP data of each mouse were converted to a z-score by subtracting the mean LFP and dividing by the standard deviation of the signal.

#### Spectral analysis

Power spectral density was computed using multi-taper methods of the NiTime Python library with a bandwidth of 1 (www.nipy.org). Power was calculated in 5 s data trunks and averaged for each animal. In case of animals with implanted microdrives, one of the channels used for spike recording was chosen for LFP analysis (usually channel 1). In case of multiple recording sessions, power spectra were obtained for each session independently and averaged to obtain one power spectrum per animal. Power spectra were normalized by frequency to account for the 1/f decay of power with frequency to allow for comparisons between different frequency bands^44^. Frequency-filtered data were obtained by applying a 2^nd^-order Butterworth band-pass filter in forward and reverse direction to avoid phase shifts. The filter was generated with the scipy.signal.butter function applied to the data via scipy.signal.filtfilt. For display, LFP data were band-pass filtered between 1 and 200 Hz. Respiration recordings were filtered between 1 and 10 Hz. Coherence was measured using scipy.signal.coherence with a sliding window of 0.5 * sampling frequency duration zero-padded to 10 * sampling frequency.

#### n:m phase-phase coupling

Phase-phase coupling analysis of PRR and theta oscillations was performed by extracting the instantaneous phase of the 1–5 Hz and 6–12 Hz frequency-filtered data from the phase component of the Hilbert transform (scipy.signal.hilbert). Joint phase histograms were obtained by plotting the binned theta phase as a function of the binned PRR phase (18 bins, corresponding to 20°/bins). The phase of the PRR signal was systematically accelerated by a factor *m* ranging from 1 to 25. For each *m*, we computed the length of the mean vector of phase differences between both phase signals, *R*_*n:m*_, *as*


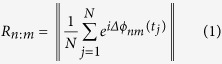


where


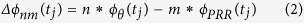


denotes the phase difference between theta and the *m*-accelerated phase of the PRR signal^25^. For each animal, the calculation was performed on 25 data segments (length 10 s) with randomly chosen starting points within the recording. Surrogate data were created by shifting the PRR phase signal by a random interval between 5 and 10 s. Averaging yielded a single *R*_*n:m*_ and surrogate plot per mouse. Pairwise statistical comparison between *R*_*n:m*_ obtained from the recording and the matched surrogate data of each animal allowed us to distinguish real from spurious phase-phase coupling^25^.

#### Frequency-amplitude coupling

Frequency-amplitude coupling was measured as described previously^29^. In brief, a set of narrow band-pass filters were applied to the LFP data to isolate PRR, theta (2–10 Hz, 1 Hz bandwidth, 1 Hz steps) and gamma frequencies (30–150 Hz, 10 Hz bandwidth, 1 Hz steps, 2^nd^ order Butterworth filter). Next, instantaneous phase of the slow modulating oscillation and envelope of the fast modulated oscillation were obtained using the amplitude and phase components of the Hilbert transform. For each pair of slow and fast oscillation, a mean gamma envelope was determined in 20° phase bins. Average gamma amplitude was normalized to the sum over all bins. The Kullback-Leibler distance^45^ was calculated as


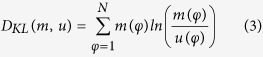


between the measured gamma envelope distribution as a function of phase *m(φ*) to a uniform distribution of identical mean value *u(φ*) with *N* being the number of phase bins. Modulation index was derived by dividing the obtained distance by the log of the bin number (modulation index of 0: no modulation, 1: maximal modulation). This procedure was applied in a non-overlapping sliding window (length: 4 s) and averaged for each mouse.

#### Extraction of theta-rich epochs

To extract epochs of significant theta activity, the root mean square (rms) of the theta-filtered LFP was computed. A threshold was defined as


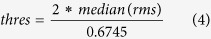


Detected time periods above the defined threshold were considered to contain significant theta activities. These time epochs comprised <2% of the total recording duration.

#### Analysis of single-unit recordings

Single unit activity was analysed with the KlustaSuite environment^46^. To extract spikes, the signals were band-pass filtered between 0.6 and 6 kHz. Spike waveforms were isolated using ‘SpikeDetect’ and automatically sorted into clusters using ‘KlustaKwik’^47^. The obtained clusters were manually controlled in the ‘KlustaViewa’ environment. Clusters containing multiple single units (evident from the lack of a >1 ms absolute refractory period in the autocorrelogram^48^) were manually split and only kept if splitting resulted in single-units with clear refractory period. Individual clusters belonging to the same unit (evident from a symmetric drop in the cross-correlogram at short time lags) were merged. To measure coupling of unit discharges to LFP oscillations, band-pass filters were applied to the LFP to extract PRR (1–5 Hz) and theta (6–12 Hz) frequency components. The phase (ranging from −π to π) at each spike time of a given unit was recorded. Rayleigh’s test for circular uniformity was used to assess significant phase-coupling. This analysis was only performed on units that discharged at least 10 spikes during the analysed immobility epochs.

### Statistical comparisons

For data that followed a normal distribution, significance was assessed with an unpaired or paired two-tailed student’s *t*-test, otherwise a two-tailed Mann-Whitney U test (independent data) or two-tailed Wilcoxon’s signed rank test (dependent data) was used. Normality was tested with a Shapiro-Wilk test. Multiple comparisons were made after applying Bonferroni correction to adjust p_critical_. Rayleigh’s test for circular uniformity was used to assess phase-phase coupling. All statistical comparisons were calculated with the python package scipy.stats at the significance level indicated.

## Additional Information

**How to cite this article:** Biskamp, J. *et al*. Organization of prefrontal network activity by respiration-related oscillations. *Sci. Rep.* **7**, 45508; doi: 10.1038/srep45508 (2017).

**Publisher's note:** Springer Nature remains neutral with regard to jurisdictional claims in published maps and institutional affiliations.

## Supplementary Material

Supplementary Figures
