# Supplementary material for: Organization of prefrontal network activity by respiration-related oscillations
Source: Sci Rep. 2017 Mar 28;7:45508. doi: 10.1038/srep45508 (PMC5368652; doi:10.1038/srep45508)
Supplement: Supplementary Figures [file srep45508-s1.pdf]

Supplementary Information

Organization of prefrontal network activity by respiration-related oscillations

Jonatan Biskamp, Marlene Bartos and Jonas-Frederic Sauer

Physiologisches Institut I, Systemic and Cellular Neurophysiology, Albert-Ludwigs-Universität Freiburg, Hermann-Herder-Straße 7, 79104 Freiburg, Germany

Supplementary Figures S1-8

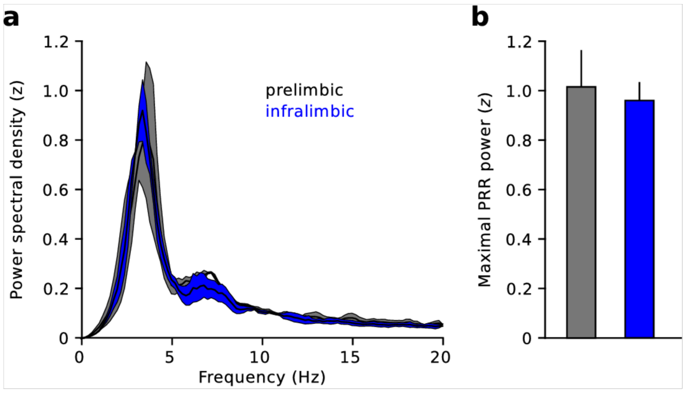

Supplementary Figure S1: Comparable spectral properties of the LFP at prelimbic and infralimbic recording sites. (**a**) Power spectral densities of LFP recordings from the prelimbic (black) and infralimbic area of the mPFC (blue) during immobility in the TST. Data were obtained sequentially from the same animals by advancing electrodes mounted on a microdrive. (**b**) Summary of maximal power in the PRR frequency range (1-5 Hz). n = 3 mice, p = 0.785, paired *t*-test. Data are mean ± sem.

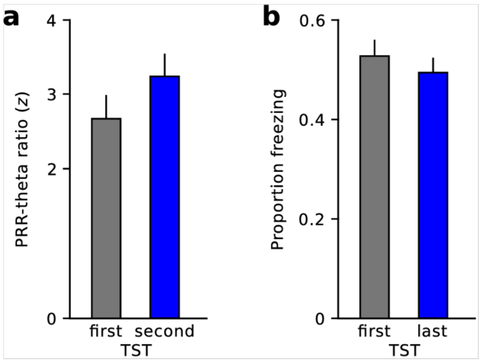

Supplementary Figure S2: Repeated exposure to TST does not alter spectral properties or behavioural responses. (**a**) Summary of PRR-theta ratio during TST immobility on two consecutive days revealed no difference in spectral properties. n = 6 mice, p = 0.107, paired *t*-test. (**b**) Summary graph showing the proportion of freezing during the first and the last TST. Last TST corresponds to the third or fourth day of TST with one TST experiment daily. n = 6 mice, p = 0.174, paired *t*-test. Data are mean ± sem.

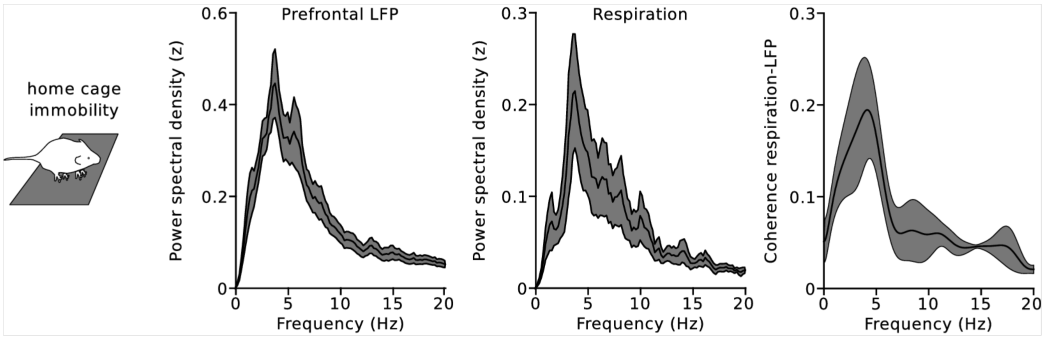

Supplementary Figure S3: PRR activities emerge during immobility in the home cage. Similar to TST recordings, power spectral density of the prefrontal LFP (left, n = 13) and respiration peaks (middle, n = 4) occur in the PRR range of 1-5 Hz. Right plot: LFP and respiration recording are maximally coherent in the PRR frequency range. n = 4 mice. Data are mean ± sem.

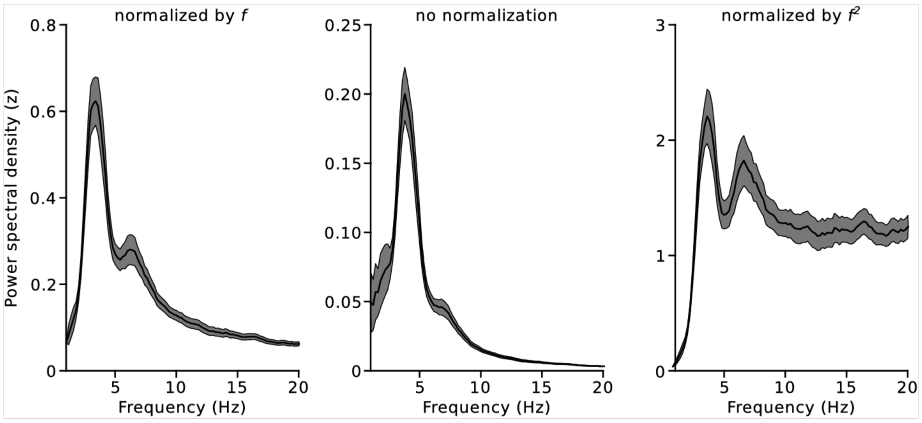

Supplementary Figure S4: Prefrontal spectral properties are maintained with different normalization methods. Average power spectra of prefrontal LFP during TST immobility normalized by multiplication with frequency *f* (left, used in the remainder of the manuscript), without normalization (middle), and normalized by multiplication with f^2 (right). Note that a prominent peak emerges at ~3.5 Hz independent of the normalization regime. n = 25 mice. Data are mean ± sem.

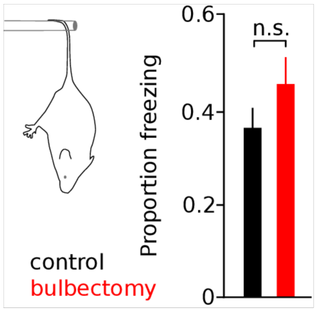

Supplementary Figure S5: Olfactory bulbectomy does not affect freezing in the TST. The bar graph summarizes the proportion freezing of control (black, 0.45 ± 0.06, tested for a sample of n = 12 mice) and bulbectomized mice (red, 0.35 ± 0.04, n = 7, p = 0.276, unpaired *t*-test). Data are mean ± sem.

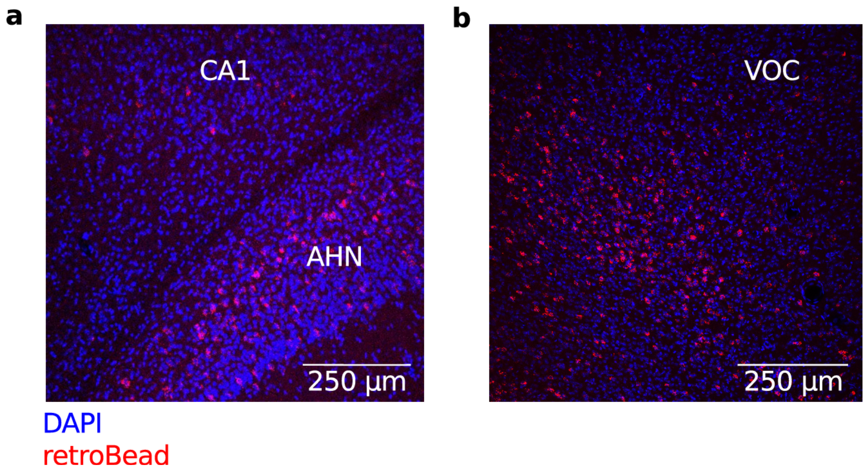

Supplementary Figure S6: Additional retrograde labeling in neurons of the amygdalo-hippocampal nucleus and ventro-orbital cortex upon tracer injection into the mPFC. (**a**) In addition to neurons in the olfactory cortices (**Fig. 2**), retroBead-labelled cells were observed in the ventral hippocampal area CA1 and in the amygdalo-hippocampal nucleus (AHN). (**b**) Labelled cells were also detected in the ventro-orbital cortex (VOC).

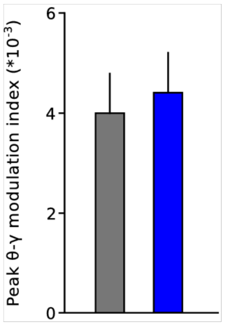

Supplementary Figure S7: Indistinguishable theta-gamma coupling in hippocampal CA1 during motion and immobility. Summary graphs shows the peak theta-gamma modulation index during TST immobility (grey) and movement (blue). N = 13 mice, p = 0.346, Wilcoxon singed-rank test with Bonferroni correction (5 comparisons, cf. **Fig. 4**, $p_{critical}$ = 0.01).

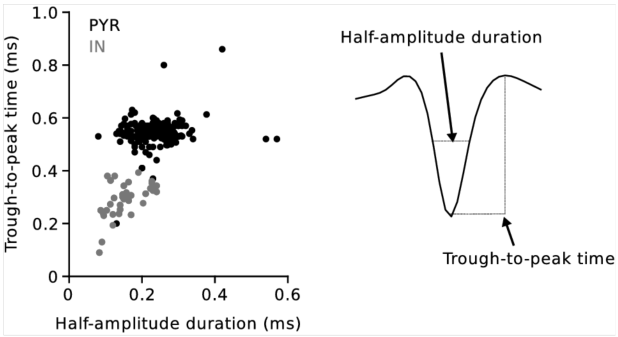

Supplementary Figure S8: Single-unit classification. Recorded single-units were identified as pyramidal neurons (PYR) or interneurons (INs) based on waveform shape. Units with a trough-to-peak duration >0.4 ms were designated as PYR while units possessing shorter trough-to-peak times were grouped as IN.
